# The impact of the 7th October war on preterm birth rates and neonatal outcomes: a retrospective comparative study from northern Israel

**DOI:** 10.1007/s00404-026-08483-3

**Published:** 2026-06-12

**Authors:** Raghda Zidan Sweid, Adi Sharabi Nov, Hana Helfgot, Steven L. Warsof, Inbar Ben-Shachar

**Affiliations:** 1 Department of Obstetrics and Gynecology, Ziv Medical Center, Safed, Israel; 2 Azrieli Faculty of Medicine, Bar-Ilan University, Ramat Gan, Israel; 3 Research wing, Ziv Medical Center, Safed, Israel; 4 Tel-Hai University of Kiryat Shmona and the Galilee, Tel Hai, Kiryat Shemona, Israel; 5 Eastern Virginia Medical School EVMS, Norfolk, VA USA

**Keywords:** Preterm birth, Wartime exposure, Labor induction, Neonatal outcomes

## Abstract

**Purpose:**

Maternal psychological stress is associated with adverse obstetric and neonatal outcomes, including preterm birth and low birthweight. Warfare represents a significant source of acute and chronic stress, yet its impact on pregnancy outcomes remains unclear. This study aimed to evaluate the association between a 6-month period of continuous wartime exposure following October 7, 2023, during the “Iron Swords” conflict and preterm birth, neonatal outcomes, and obstetric management.

**Methods:**

This retrospective cohort study included all deliveries at ZIV Medical Center during the 6-month conflict period, compared with a pre-war cohort from 2021 until October 6, 2023. The primary outcome was preterm birth (< 37 weeks). Secondary outcomes included early preterm birth (< 34 weeks), labor induction, mode of delivery, maternal complications, and neonatal outcomes. Multivariate logistic regression was used to identify independent predictors of preterm birth.

**Results:**

A total of 699 conflict-period deliveries were compared with 7821 pre-war deliveries. Preterm birth rates were similar (< 37 weeks: 6.6% vs. 5.5%; < 34 weeks: 2.1% vs. 1.5%). Labor induction was markedly lower during the conflict (oxytocin 23.7% vs. 38.7%; cervical ripening balloon 3.3% vs. 7.7%; prostaglandins 4.1% vs. 6.5%), accompanied by reduced postpartum hemorrhage (7.9% vs. 12%). Neonatal outcomes, including birthweight distribution, NICU admissions, and survival, were comparable or slightly improved.

**Conclusion:**

Prolonged wartime exposure was not associated with increased rates of preterm birth or adverse neonatal outcomes. Lower rates of obstetric intervention were observed during the conflict period and coincided with lower rates of maternal complications, although causality cannot be established.

## What does this study adds to the clinical work

**Table:** 

In our cohort, wartime exposure during the October 7th conflict was not associated with significant changes in preterm birth rates or neonatal outcomes. Further studies are needed to evaluate the impact of armed conflicts on maternal and neonatal health in different populations and settings.	

## Introduction

Maternal psychological stress has long been recognized as a significant risk factor for adverse obstetric and neonatal outcomes [1–3]. Extensive research indicates that exposure to acute or chronic stressors during pregnancy can activate the maternal hypothalamic–pituitary–adrenal axis, increasing placental corticotropin-releasing hormone and cortisol production, which may prematurely trigger the biological pathways of labor [4]. Consequently, high maternal stress is frequently associated with increased rates of preterm birth (PTB) and low birthweight (LBW) [3, 4].

Warfare and national conflict are profound sources of environmental and psychological stress. Studies from diverse conflict settings have shown that pregnant women exposed to violence are at increased risk of adverse pregnancy outcomes. In particular, exposure to terrorist attacks and civil unrest has been associated with higher rates of PTB, with risk increasing alongside exposure intensity [3, 5, 6].

Furthermore, wartime exposure has also been associated with increased maternal psychological morbidity, including postpartum depression, anxiety, and impaired maternal–infant bonding [7, 8].

In the Israeli context, previous studies investigating the impact of security escalations have shown that exposure to continuous missile threats is associated with increased risks of preterm premature rupture of membranes, gestational diabetes mellitus, and postpartum hemorrhage [4, 9, 10]. However, Gluck et al. found that short-term exposure to military-induced stress during pregnancy was not associated with an increased risk of pregnancy complications, regardless of stress severity or gestational age at the time of exposure [11].

On October 7, 2023, the “Iron Swords” war began in southern Israel and rapidly extended to the northern region, exposing the civilian population to sustained missile threats, displacement, and ongoing psychological stress. This conflict represents a unique clinical setting due to its prolonged duration and the close proximity of healthcare facilities to active combat zones. ZIV Medical Center, located in Safed, serves a population directly affected by these conditions, offering a valuable opportunity to assess the impact of prolonged wartime exposure on maternal and neonatal outcomes.

This study aimed to assess whether a defined 6-month period of wartime exposure following October 7, 2023, during the “Iron Swords” conflict, influenced the rates of preterm birth and neonatal morbidity compared with the pre-war control period. It also examined associated changes in obstetric management, including labor induction and mode of delivery.

### Materials and methods

This retrospective cohort study was conducted at ZIV Medical Center, a referral hospital in northern Israel that was directly exposed to wartime conditions. We compared all deliveries that occurred during the 6-month period following October 7, 2023, representing the acute phase of the war, with deliveries during 2021 until October 6, 2023, which served as a pre-war reference cohort. All singleton and twin pregnancies delivered at the institution during the study periods were eligible for inclusion. Twin pregnancies were included in all analyses, and pregnancy type (singleton vs. twin) was included as a covariate in the multivariable regression model.

Pregnancies with unknown gestational dating and cases in which the patient was admitted but delivered at another institution were excluded.

Maternal demographic and obstetric data were extracted from the electronic medical records. Variables collected included the type of pregnancy (singleton or twin), gestational age at delivery, evacuation from home during the conflict period, mode of delivery (spontaneous vaginal delivery, instrumental vaginal delivery, or cesarean delivery), indication for cesarean delivery, use of labor induction methods (oxytocin, prostaglandins, cervical ripening balloon), use of epidural analgesia, and intrapartum complications such as postpartum hemorrhage, fever, and severe perineal laceration. Neonatal data included birthweight, 5-min Apgar score, need for neonatal intensive care unit (NICU) admission, respiratory distress syndrome, necrotizing enterocolitis, sepsis, intraventricular hemorrhage, hypoglycemia, and neonatal mortality.

The primary outcome was preterm birth before 37 weeks of gestation. Secondary outcomes included early preterm birth (< 34 weeks), birthweight distribution, NICU admission, 5-min Apgar score < 7, mode of delivery, labor induction rates, and maternal intrapartum complications.

To assess the potential for selection bias related to evacuation status during the conflict period, demographic and obstetric characteristics were compared between women who were evacuated from their homes and delivered at ZIV and those who remained in the region. Variables assessed included maternal age, parity, pregnancy type, maternal comorbidities (including hypertensive disorders, gestational diabetes mellitus (GDM), and pregestational diabetes mellitus (DM)), as well as delivery characteristics and obstetric outcomes. No statistically significant differences were observed between the groups across the analyzed variables (Table 1), suggesting overall similarity between evacuated and non-evacuated women within the study population.

**Table 1:** Maternal and pregnancy characteristics of the conflict-exposed group

Evacuation from home			
Variables	No (*n* = 664)	Yes (*n* = 35)	*p* value
*Maternal age group, yr. (%)*			
19–25	21.7	0.0	0.658
26–40	72.3	100	
41 +	6.0	0,0	
Nulliparous (%)	26.1	25.7	0.964
*Type of pregnancy (%)*			
Singleton	97.9	100	0.636
Twins	2.1	0,0	
*Maternal risk factors (%)*			
DM	1.4	2.9	0.466
GDM	2.1	2.9	0.766
PE	2.0	0.0	0.644
*Type of delivery (%)*			
Vaginal	76.8	80.0	0.600
Cesarean section	19.7	14.3	
Vacuum extraction delivery	3.5	5.7	
Epidural (%)	49.5	57.1	0.381

*DM* diabetes mellitus, *GDM* gestational diabetes mellitus, *PE* preeclampsia

### Statistical analysis

Categorical variables were summarized as percentages and compared using Pearson’s Chi-square test. A multivariate logistic regression model was calculated for the correlation between preterm birth (< 37 weeks) and maternal complications (DM, GDM, hypertension disorders), type of pregnancy (singleton/twin), nulliparity, previous PTB, and evacuation from home during wartime, with adjustment to maternal age. A *p* value ≤ 0.05 was considered statistically significant. Statistical analyses were performed using SPSS version 31 (IBM).

The local institutional review board approved the study protocol (# ZIV-0025-24).

## Results

A total of 699 deliveries occurred during the conflict period, compared with 7821 deliveries in the pre-war control period. Maternal age distribution and parity were similar between groups. Nulliparity was slightly higher in the conflict-exposed group (26.0% vs. 24.1%, *p* = 0.160). The proportion of singleton and twin pregnancies was identical in both groups (98% singleton, 2% twins, *p* = 0.936).

Maternal risk factors differed between groups: the conflict-exposed group had a higher rate of DM (1.4% vs. 0.6%, *p* = 0.004) but a lower rate of GDM (2.1% vs. 5.3%, p < 0.001). Rates of preeclampsia (PET) and hypertension were similar between groups (Table 2).

**Table 2:** Maternal and pregnancy characteristics

Variables	Conflict-exposed group (*n* = 699)	Control group (*n* = 7821)	*p* value
*Maternal age group, yr. (%)*			
19–25	21.3	16.5	0.160
26–40	72.8	78.8	
41 +	5.9	4.7	
Nulliparous (%)	26.0	24.1	0.297
*Type of pregnancy (%)*			
Singleton	98.0	98.3	0.0.936
Twins	2.0	1.7	
*Maternal risk factors (%)*			
DM	1.4	0.6	0.004
GDM	2.1	5.3	< 0.001
PE	1.9	1.2	0.100
Hypertension	0.1	0.2	0.609

*DM* diabetes mellitus, *GDM* gestational diabetes mellitus, *PE* preeclampsia

Mode of delivery was comparable across groups, with approximately 77% vaginal deliveries, 19% cesarean sections, and 3–4% vacuum-assisted deliveries (*p* = 0.686). Epidural use was also similar (48.8% vs. 46.8%, *p* = 0.100).

No significant differences were observed in gestational age distribution. Rates of preterm birth (< 37 weeks) were 6.6% in the conflict-exposed group and 5.5% in controls (*p* = 0.258).

Labor induction patterns differed significantly between the groups. Use of a cervical ripening balloon was lower in the conflict-exposed group (3.3% vs. 7.7%, *p* < 0.001), as was oxytocin induction (23.7% vs. 38.7%, p < 0.001) and the prostaglandin use (4.1% vs. 6.5%, *p* = 0.012).

Perineal lacerations of any level showed no significant differences between the two groups. Postpartum hemorrhage occurred less frequently in the conflict-exposed group (7.9% vs. 12%, *p* = 0.001), with no differences in postpartum hysterectomy (Table 3).

**Table 3:** Delivery outcomes

Variables	Conflict-exposed group (*n* = 699)	Control group (*n* = 7821)	*p* value
*Type of delivery (%)*			
Vaginal	77.0	77.3	0.686
Cesarean section	19.4	18.6	
Vacuum extraction delivery	3.6	4.1	
*Gestational age, weeks (%)*			
24–33^+6^	2.1	1.5	0.475
34–36^+6^	4.4	4.1	
37–38^+6^	25.6	27.0	
39–40^+6^	54.2	55.2	
41 +	13.7	12.2	
Preterm birth (%)	6.6	5.5	0.258
Epidural (%)	48.8	46.8	0.100
*Labor induction (n, %)*			
Prostaglandins	4.1	6.5	0.012
Cervical ripening balloon	3.3	7.7	< 0.001
Oxytocin	23.7	38.7	< 0.001
*Laceration (%)*			
None, Levels 1, 2	89.3	88.8	0.152
Level 3 + 4	0.1	0.8	
Episiotomy	10.6	10.8	
Postpartum hysterectomy (%)	0	0.05	0.998
Postpartum hemorrhage (%)	7.9	12.0	0.001

Neonatal survival was similar (alive at discharge: 99.5% vs. 99.2%, *p* = 0.536).

The birthweight distribution revealed a higher proportion of infants with normal birthweight (2500–4,000 g) in the conflict-exposed group (90.6% vs. 87.2%, *p* = 0.050). Extremely low birthweight (< 1500 g) was slightly lower in the conflict-exposed group (0.7% vs. 0.8%).

NICU admissions were comparable between the groups (7.0% vs. 8%, *p* = 0.323) and no significant differences were noted in 5-min Apgar scores < 7 (0.1% vs. 0.2%, *p* = 0.690) (Table 4).

**Table 4:** Neonatal outcomes

Variables	Conflict-exposed group (*n* = 699)	Control group (*n* = 3182)	*p* value
*Last status of infant (%)*			
Alive	99.5	99.2	0.536
Dead	0.5	0.4	
*Birthweight, gr. (%)*			
< 1500	0.7	0.8	0.050
1501–2000	1.4	1.5	
2001–2500	4.1	5.1	
2501–4000	90.6	87.2	
4001–4500	3.2	5.4	
APGAR 5 min < 7 (%)	0.1	0.2	0.690
NICU (%)	7.0	8.0	0.323

*NICU* neonatal intensive care unit

A multivariable logistic regression analysis was performed to identify independent predictors of preterm birth (PTB) (Table 5). Twin gestations were associated with a significantly increased risk of PTB compared with singleton pregnancies (OR = 21.61, 95% CI 6.95–67.22, *p* < 0.001). Women who were evacuated from their homes during the wartime period had a fourfold higher risk of PTB compared with non-evacuated women (OR = 4.03, 95% CI 1.53–10.64, *p* < 0.01). Nulliparity was also associated with a higher risk of PTB compared with multiparity; however, this association was of borderline statistical significance (OR = 1.84, 95% CI 0.94–3.62, *p* = 0.076).

**Table 5:** Multivariate logistic regression model for the correlation between preterm birth (< 37 weeks) and maternal complication (DM, GDM, Hypertensive disorders), type of pregnancy (singleton/twin), nulliparity, previous PTB and evacuation from home during wartime with adjustment to maternal age

Variables	Values	OR	95% CI	*p*
Maternal age	Continuous	0.99	0.94–1.05	0.730
Previous PTB	Yes vs. no	2.06	0.54–7.83	0.289
DM	Yes vs. no	3.19	0.58–17.67	0.184
GDM	Yes vs. no	0.80	0.27–67.22	0.696
Hypertensive disorders	Yes vs. no	2.37	0.47–12.01	0.297
Nulliparous	1 vs. others	1.84	0.94–3.62	0.076
Type of pregnancy	Twin vs. singleton	21.61	6.95–67.22	< 0.001
Evacuation from home	Yes vs. no	4.03	1.53–10.64	0.005

*OR* odds ratios, *CI* confidence interval, *DM* diabetes mellitus, *GDM* gestational diabetes mellitus, *PE* preeclampsia, *PTB* preterm birth

No significant associations were observed between maternal complications—including hypertensive GDM, or a history of previous PTB—and the risk of PTB before 37 weeks of gestation.

## Discussion

Our study demonstrated that exposure to wartime conditions during the 6-month period following the October 7th conflict was not associated with a detectable increase in preterm birth rates. Despite repeated missile alerts, population displacement, and widespread psychological stress, both overall preterm birth (< 37 weeks) and early preterm birth (< 34 weeks) remained comparable to the pre-war reference period. These findings suggest that prolonged environmental stress at the population level does not necessarily translate into higher rates of preterm delivery.

Our results are consistent with a previous Israeli study suggesting that short-term exposure to military-related stress during pregnancy may not significantly increase the risk of adverse obstetric outcomes [11]. However, studies conducted in other conflict settings have reported increased rates of preterm birth and low birthweight [12, 13]. These discrepancies likely reflect the complex interaction between psychological stress, population characteristics, access to healthcare, and adaptive responses within healthcare systems during crisis situations.

A notable finding in our cohort was the significant reduction in labor induction during the conflict period, including decreased use of oxytocin, cervical ripening balloons, and prostaglandins. This reduction may reflect operational constraints during wartime, as the maternity department was relocated to a protected facility with limited labor and hospitalization capacity. In addition, changes in regional referral patterns and population displacement during the conflict period may have influenced patient flow and obstetric management practices. Pregnant women may have delayed non-urgent hospital presentations because of security concerns, while clinicians may have adopted a more conservative approach to obstetric interventions during periods of heightened uncertainty.

Interestingly, the reduction in obstetric interventions coincided with improved maternal outcomes, including lower rates of postpartum hemorrhage and a trend toward fewer severe perineal lacerations. Previous observational studies have suggested that labor induction or augmentation may be associated with an increased risk of postpartum hemorrhage and perineal trauma [14–18]. However, given the retrospective observational design of the present study, these findings should be interpreted with caution, and no causal relationship between the reduction in obstetric interventions and the observed improvement in maternal outcomes can be inferred.

Neonatal outcomes remained stable throughout the study period. The proportion of infants born within the normal birthweight range was slightly higher during the conflict period. Although maternal stress has frequently been associated with fetal growth restriction and adverse neonatal outcomes in prior studies [12, 13], such patterns were not observed in our cohort. These findings suggest that other protective factors, including stable access to obstetric care and adaptive healthcare system responses, may have mitigated the potential impact of environmental stressors.

In the multivariable analysis, twin gestation and evacuation during the conflict period were independently associated with preterm birth. In contrast, maternal complications and a history of previous PTB were not independently associated with PTB. These findings differ from those reported in the existing literature and may be explained by limited statistical power, as reflected by the wide confidence intervals, or by differences in clinical management and population characteristics during the study period.

Several contextual factors should be considered when interpreting these findings. The marked reduction in the number of deliveries during the conflict period likely reflects large-scale population displacement from northern Israel. Pregnant individuals who relocated may have delivered at other institutions and were therefore not captured in our dataset, introducing a potentially important source of selection bias. Consequently, the women who remained in the region may have differed systematically from those who relocated with respect to health status, obstetric risk profiles, healthcare utilization patterns, or access to medical care. Although no significant differences were identified between evacuated and non-evacuated women across the analyzed demographic and obstetric variables, the possibility of residual and unmeasured confounding cannot be excluded. These factors may limit both the internal validity and the generalizability of the study findings.

In addition, exposure to wartime-related stress was inferred solely based on the study period rather than measured directly at the individual level using validated psychological instruments. This represents a major limitation of the study and may have resulted in substantial exposure misclassification. Individual stress perception, coping capacity, proximity to missile attacks, duration of exposure, and degree of conflict-related disruption likely varied considerably among participants and could not be adequately captured using the available data. Consequently, the study may not fully reflect the true relationship between maternal psychological stress and obstetric outcomes.

This study also has several strengths. It reflects real-world data from a population directly exposed to prolonged wartime conditions and includes a large pre-war cohort from the same institution, thereby reducing variability in clinical protocols and obstetric management practices. In addition, the inclusion of both maternal and neonatal outcomes enabled a comprehensive evaluation of the potential impact of conflict exposure on pregnancy outcomes. The hospital’s location in northern Israel provided a unique opportunity to evaluate a population experiencing sustained exposure to conflict-related stressors.

Nevertheless, several additional limitations should be acknowledged. Despite the inclusion of all deliveries occurring at our institution during the study period, the wartime cohort remained relatively small, which may have limited the statistical power to detect clinically meaningful differences in less common outcomes, including early preterm birth, neonatal morbidity, and severe maternal complications. Therefore, negative findings should be interpreted cautiously, as the study may have been underpowered for certain outcomes. Finally, this was a single-center study conducted at a tertiary medical center, and the findings may not be generalizable to other healthcare settings or populations.

In conclusion, 6 months of exposure to wartime conditions following the October 7 conflict were not associated with an increased risk of preterm birth or neonatal morbidity in our population. Despite considerable environmental and psychological stressors, obstetric and neonatal outcomes remained largely stable. Although lower rates of obstetric interventions were observed during the conflict period, the retrospective observational design of the study precludes any conclusions regarding the safety or effectiveness of a more conservative approach to labor management.

Future multicenter studies incorporating direct assessments of maternal psychological stress and including displaced populations are warranted to further clarify the complex relationship between conflict exposure, healthcare utilization, and pregnancy outcomes.

## Data Availability

No datasets were generated or analyzed during the current study.
